# Endocannabinoid System Regulation in Pyometra-Affected and Healthy Canine Uteri

**DOI:** 10.3390/vetsci12100934

**Published:** 2025-09-25

**Authors:** Anıl Gürkan Aksu, Volkan Ferahoğlu, Fatih Büyükbudak, Isil Unaldi, Aykut Gram, Murat Fındık, Serhan Serhat Ay

**Affiliations:** 1 Department of Obstetrics and Gynecology, Faculty of Veterinary Medicine, Ondokuz Mayıs University, Samsun 55500, Türkiye; a.gurkanaksu@gmail.com (A.G.A.); volkan.ferahoglu@omu.edu.tr (V.F.); fatih.buyukbudak@omu.edu.tr (F.B.); mfindik@omu.edu.tr (M.F.); 2 Department of Biostatistics and Medical Informatics, Faculty of Medicine, Ondokuz Mayıs University, Samsun 55500, Türkiye; isil.unaldi@omu.edu.tr; 3 Department of Histology and Embryology, Faculty of Veterinary Medicine, Erciyes University, Kayseri 38039, Türkiye; aykutgram@erciyes.edu.tr

**Keywords:** dog, endocannabinoid system, pyometra

## Abstract

Pyometra is a frequent and life-threatening reproductive disorder in bitches, characterized by profound immune and inflammatory responses within the uterus. The endocannabinoid system (eCS) is a key modulator of immune regulation, tissue homeostasis, and inflammation; however, its role in canine uterine physiology and pathology remains unexplored. This study aimed to characterize the presence and regulation of eCS components in the uterus of healthy and pyometra-affected dogs. Twenty-eight bitches were categorized into four groups: closed-cervix pyometra (CP; *n* = 7), open-cervix pyometra (OP; *n* = 7), diestrus (DE; *n* = 7), and anestrus (AE; *n* = 7). Uterine tissues were obtained by ovariohysterectomy. Serum progesterone, anandamide (AEA), and 2-arachidonylglycerol (2-AG) concentrations were quantified, while the uterine expression of cannabinoid receptors (CB1 and CB2) was assessed using real-time PCR and localized by immunohistochemistry (IHC). Serum AEA levels were significantly reduced in CP compared with AE (*p* = 0.017), whereas 2-AG differences did not reach significance (*p* = 0.072). Both CB1 and CB2 were consistently expressed across all groups, with IHC revealing receptor-specific patterns within uterine compartments. Collectively, these findings demonstrate for the first time that the canine uterus possesses a functional eCS, and that its modulation is linked to reproductive physiology and pyometra-associated inflammatory processes.

## Simple Summary

Pyometra is a common and potentially life-threatening uterine infection in female dogs, characterized by strong immune and inflammatory reactions. The endocannabinoid system (eCS) is known to regulate immunity and inflammation in the body, but its role in the canine uterus has not been studied. In this study, we examined uterine tissues from pyometra-affected and healthy dogs to investigate the presence of eCS components. We analyzed endocannabinoids in the serum and evaluated their receptor expression in the uterus. Our results show that the canine uterus has a functional eCS, with specific patterns of receptor expression, and that changes in this system are associated with reproductive status and inflammatory responses during pyometra. These findings provide new insights into uterine physiology and potential therapeutic targets for canine pyometra.

## 1. Introduction

Pyometra is one of the most common and life-threatening reproductive disorders in intact bitches, often resulting in systemic illness and, in severe cases, sepsis. It typically affects middle-aged to older animals during the luteal phase. Pyometra is characterized by bacterial infection of the uterus and accumulation of purulent material within the uterine lumen [1]. It is also considered a spontaneous clinical model for sepsis due to pathological and immunological parallels with human infection-associated disorders [2]. Despite its prevalence, the molecular mechanisms that predispose to or exacerbate pyometra remain incompletely understood [3].

The pathogenesis of pyometra reflects a multifactorial interaction between endocrine regulation, uterine tissue changes, and bacterial infection. Progesterone (P4)-mediated immunosuppression and structural changes in the endometrium create a uterine environment conducive to bacterial persistence and inflammation. Prolonged exposure to P4 suppresses neutrophil migration, downregulates cytokine production, and enhances glandular secretions [1]. Over time, this hormonal influence can induce endometrial changes such as cystic endometrial hyperplasia (CEH) and pseudoplacentational endometrial hyperplasia (PEH), both of which may create a microenvironment favorable for bacterial colonization [4].

*Escherichia coli (E. coli)* is the most frequently isolated pathogen from pyometra cases and one of the major sources of lipopolysaccharide (LPS) [5]. Transcriptomic studies of pyometra-affected uteri demonstrated changes, with eight hundred genes involved in chemokine signaling, complement activation, and antibacterial defense being upregulated [2]. One of them is gene-encoding Toll-like receptor-4 (TLR-4) [5]. *Escherichia coli* and its LPS activate TLR-4 signaling initiating inflammatory cascades that lead to pyometra pathogenesis on uterine epithelial, stromal, and resident immune cells. Toll-like receptor-4 engagement triggers chemokine production upregulates cyclooxygenase-2 (COX-2) and stimulates prostaglandin (PG) synthesis [5]. The dysregulation of these processes amplifies tissue injury and contributes to systemic inflammatory responses characteristic of sepsis.

The endocannabinoid system (eCS) is defined as a neuromodulator network, comprising bioactive lipids, receptors, and metabolic enzymes. It has emerged as a potential but unexplored regulatory pathway [6,7]. The eCS includes endocannabinoids (ECBs) such as anandamide (AEA) and 2-arachidonoylglycerol (2-AG), their receptors CB1 and CB2, and metabolic enzymes including N-acyl-phosphatidylethanolamine phospholipase D (NAPE-PLD), fatty acid amide hydrolase (FAAH), diacylglycerol lipases (DAGL) and monoacylglycerol lipase (MAGL) [6,7]. Endocannabinoid system mediators are synthesized exclusively on demand and are not stored [8]. Anandamide mediates its biological effects predominantly via CB1, whereas 2-AG signals primarily through CB2 or through both CB1 and CB2, with the potential of both ECBs activating either receptor depending on the tissue-specific context [9]. Cannabinoid receptor 1 is primarily expressed in the nervous system, whereas CB2 predominates in immune cells such as macrophages and lymphocytes, where it regulates inflammatory signaling [6].

In addition to its role in homeostasis, studies in humans and rodents show that eCS has been implicated in both reproductive physiology and pathology [6,10] as well as the immune response during sepsis [11]. Endocannabinoid system components are expressed in ovarian and uterine tissues, influencing ovulation, fertilization, and implantation [7]. Disrupted ECB signaling has been linked to infertility [6] and endometriosis [12]. In sepsis models, CB1 blockade and CB2 modulation affect blood pressure, thermoregulation, inflammatory cytokine release, and survival, highlighting context-dependent immune regulation by eCS mediators [11].

The immunomodulatory function of the eCS may be relevant to pyometra. Interactions between the eCS and TLR-4/LPS pathways suggest that ECBs may modulate inflammation in infection-associated reproductive disorders [13]. Macrophages, principal sources of ECBs, are activated by TLR signaling, modifying both CB expression and ECB levels [13]. Lipopolysaccharide exposure in sepsis models increases CB1 expression while reducing CB2, indicating receptor-specific reprogramming [14]. Elevated ECB concentrations can suppress LPS-induced pro-inflammatory mediator release in a CB1/2-dependent manner [15]. We believe that this positions the eCS at a critical intersection of hormonal, immune, and metabolic pathways relevant to pyometra pathophysiology.

Despite research in humans and rodents, eCS expression in the canine uterus remains uncharacterized, representing a significant knowledge gap. Most canine eCS studies are limited to non-reproductive tissues, including the skin [16], gastrointestinal tract [17], hair follicles [18], salivary glands [19], and central nervous system [20]. To date, no systematic investigation of eCS components in the canine uterus has been performed, despite the high prevalence of pyometra and its relevance as a model for sepsis. Considering that pyometra is one of the major causes of morbidity and mortality in bitches [1], and a natural model for studying infection-driven reproductive disease [2], characterization of uterine eCS components may provide novel insights into disease mechanisms and potential therapeutic targets.

Based on these considerations, we hypothesize that the canine uterus expresses key eCS components. The eCS activity may modulate the uterine immune response to bacterial infection by interacting with TLR-4/LPS signaling under P4-dominated endocrine conditions. Accordingly, the objective of this study was to systematically characterize the expression and localization of CB1, CB2 in the uteri of pyometra-affected and healthy bitches. To the best of our knowledge, this represents the first comprehensive evaluation of the ECS in canine uterine tissue, with implications for both veterinary reproductive pathology and broader understanding of infection-associated inflammation.

## 2. Materials and Methods

### 2.1. Animals and Groups

The study was carried out on the uterine tissue and blood samples of 28 dogs. The dogs included in the study were of different breeds and body weights (mean 13.75 kg; range 7.3–32.5 kg) and of a mean age of 5.35 years (range 1–12 years) and were admitted to the Animal Hospital of Ondokuz Mayıs University Veterinary Faculty. Written informed consent was obtained from all owners after providing detailed information about the study. All procedures were approved by the Local Ethics Committee for Animal Experiments of Ondokuz Mayıs University (HADYEK: 2022/47) approved on 22 September 2022.

Uterine tissues and blood samples were obtained by ovariohysterectomy from pyometra-affected dogs (*n* = 14) and healthy dogs (*n* = 14). Clinical diagnosis of pyometra or estrous cycle stage was established based on anamnesis, physical examination, hematology, serum biochemistry, vaginal cytology, vaginoscopy, transabdominal ultrasonography, and serum P4 concentrations, and group assignment was subsequently validated by histomorphological evaluation after surgery.

In the pyometra group, inclusion criteria were systemic signs (lethargy, depression, anorexia, vomiting, diarrhea, polydipsia, polyuria), mucopurulent or serosanguineous vaginal discharge, ultrasonographic evidence of uterine distension with anechoic to hyperechoic fluid and cystic endometrial changes suggestive of concurrent cystic endometrial hyperplasia, and serum P4 > 10 ng/mL. To ensure homogeneity, all cases met the criteria for systemic inflammatory response syndrome determined by Hauptman et al. 1997 [21]. Dogs with vaginal discharge were assigned to the closed-cervix subgroup (CP; *n* = 7), whereas those without discharge were classified as open-cervix (OP; *n* = 7).

The second group consisted of healthy bitches undergoing elective OHE, confirmed to have no signs of systemic and reproductive disorders. Based on vaginal cytology and serum P4 concentrations, they were further divided into diestrus (DE; *n* = 7; predominance of intermediate/parabasal cells, P4 > 10 ng/mL) and anestrus (AE; *n* = 7; predominance of parabasal/small intermediate cells, P4 < 1 ng/mL) subgroups.

### 2.2. Blood Sampling and Measurements of Progesterone and Endocannabinoids

Blood samples were collected from the cephalic antebrachial vein of each animal into an EDTA tube and a gel separator tube (BD Vacutainer^®^, Becton Dickinson, Franklin Lakes, NJ, USA) just before surgery. The samples were centrifuged at 5000 rpm for 10 min (M615M, Elektro-mag, İstanbul, Türkiye) and the obtained sera were stored at −20 °C until P4, AEA and 2-AG measurements were performed.

Serum P4 measurements were performed by the electrochemiluminescence immunoassay using a Cobas Modular E170 fully automatic analyzer^®^ (Roche Diagnostic, Rotkreuz, Switzerland) and as previously published [22] at the laboratory of the TÜRKAK-accredited (TS EN ISO/IEC 17025:2005) Düzen Laboratories Group, Ankara, Türkiye. The average intra- and inter-assay coefficients of variation were 1.4% and 2.9%, respectively.

Anandamide and 2-AG were analyzed by liquid chromatography-electrospray ionization-tandem mass spectrometry (LC-ESI-MS/MS) using a Shimadzu LC-20AXR system coupled with a tandem mass spectrometer (Shimadzu 8030 MS/MS). The mass spectrometric detection was operated in the positive electrospray ionization and multiple reaction monitoring (MRM) mode.

Separations were carried out using GLS-ODS-4 C_18_ (50 × 3.0 mm; 2.1 μm) at a flow rate of 0.3 mL/min and at 40 °C. A gradient elution program with mobile phase A [0.1% (*v*/*v*) formic acid in water] and B [0.1% (*v*/*v*) formic acid in acetonitrile] was performed. The sample injection volume was 20 μL and the analytical run time was 10 min. The ESI-MS/MS conditions were as follows: interface voltage, 4.5 kV; Q1 pre-rod bias voltage, −13 V; Q3 pre-rod bias voltage, −23 V; collision energy, −14 eV; nebulizer gas flow rate, 3 mL/min; drying gas flow rate, 15 L/min; desolvation line temperature, 250 °C; and heat block temperature, 400 °C. The transitions 344.30 → 203.10 *m*/*z* and 379.00 → 287.20 *m*/*z* were used to monitor AEA and 2-AG, respectively.

Polymeric (Strata X, 30 mg/1 mL) solid phase cartridges were used. The 20 µL serum samples were diluted to 1 mL with ultrapure water and prepared for analysis by following the solid phase extraction (SPE) procedure using acetonitrile and methanol. Calibration solutions containing AEA and 2-AG standards at increasing concentrations (1–1000 ng/mL) and an internal standard (ACPA) at a constant concentration were prepared under the same SPE conditions as the samples and analyzed by the LC-MS/MS method. As a result of the analyses, calibration curves were created and the amounts of AEA and 2-AG in the serum samples were calculated using the equations of these calibration curves.

### 2.3. Surgery Procedure, Tissue Sampling and Tissue Processing

Uterine tissues were collected via ventral midline ovariohysterectomy under general anesthesia, a standard veterinary procedure. Anesthesia was induced with propofol (1%, 200 mg/20 mL, IV; Polifarma, Tekirdağ, Türkiye), followed by endotracheal intubation and maintenance with isoflurane (Isoflurane USP^®^, Adeka, Samsun, Türkiye). Intravenous fluids (Lactated Ringer’s Solution, 10 mL/kg/hour, Polifarma, Tekirdağ, Türkiye) and a constant-rate infusion of morphine HCl (Osel, İstanbul, Türkiye), ketamine (Ketasol %10^®^, İnterhas, Ankara, Türkiye), and lidocaine HCl (Jetokain^®^, Adeka, Samsun, Türkiye) were administered intraoperatively (10 mL/kg/hour). Postoperative care included fluids, antimicrobial therapy (cefazolin, 30 mg/kg/day, IM, Eqizolin^®^, Tüm ekip, İstanbul, Türkiye), and analgesia (meloxicam, 0.2 mg/kg, SC, Ipm, İstanbul, Türkiye), with supportive treatments tailored to the needs of each animal.

Immediately after surgery, a swab sample was taken under sterile conditions from the uterine lumen of each dog for microbiological evaluation and those showing bacterial growth were evaluated as cases of pyometra. Two tissue samples (1 × 1 cm) were taken from the middle part of the left uterine horn: One was fixed in 4% buffered formal overnight at +4 °C for subsequent paraffin embedding, histomorphological evaluation and further immunohistochemistry procedures; and the other was frozen at −196 °C and stored at −80 °C for further analysis by quantitative PCR (RT-qPCR).

### 2.4. RT-qPCR Analysis

Total RNA was extracted from uterine tissue using TRIzol reagent (TRIzol^®^, Invitrogen, Carlsbad, CA, USA) according to the manufacturer’s instructions. RNA concentrations were assessed with a NanoDrop™ 2000C spectrophotometer (Thermo Fisher Scientific, Wilmington, DE, USA) and standardized to 1000 ng for reverse transcription. Reverse transcription was using the iScript™ cDNA Synthesis Kit (Bio-Rad, Hercules, CA, USA). cDNA samples were stored at –20 °C until analysis. Primers for the target genes (CB1 and CB2) and reference genes (KDM4A, PTK2, and EIF4H) were designed using Primer Express 3.0 (Applied Biosystems, Thermo Fisher, Wilmington, DE, USA) and synthesized by Macrogen-Oligo (Seoul, Republic of Korea) (Table 1). Quantitative real-time PCR was performed with SsoAdvanced™ SYBR^®^ Green Supermix (Bio-Rad, Hercules, CA, USA) on a CFX^®^96 Real-Time PCR system (Bio-Rad, Hercules, CA, USA), with each sample, including negative controls, measured in triplicate. Relative mRNA expression was calculated using the comparative Ct (ΔΔCt) method, normalizing target gene Ct values to the geometric mean of reference genes and expressed as 2^−ΔΔCt^.

### 2.5. Immunohistochemical Staining

Immunolocalization of CB1 and CB2 in uterine tissue was performed using the standard streptavidin-biotin immunoperoxidase method. Tissue samples from bitches with pyometra during DE and AE were fixed in 4% formalin, embedded in paraffin, and sectioned at 2–3 µm thickness (Leica RM 2125Rt, Leica Biosystems, Deer Park, TX, USA). Sections were mounted on SuperFrost Plus slides (Menzel-Glaeser, Braunschweig, Germany), deparaffinized, rehydrated through graded ethanol, and subjected to antigen retrieval in 10 mM sodium citrate buffer (pH 6.0) in a microwave (600 W, 15 min). Endogenous peroxidase activity was quenched with 0.3% hydrogen peroxide in methanol for 20 min, and non-specific binding was blocked with Ultra V Block (Thermo Fisher, Wilmington, DE, USA) for 5 min at room temperature. Sections were incubated overnight at 4 °C with primary antibodies (Table 2). Negative controls included isotype-matched nonimmune IgGs and sections incubated with PBS without primary antibodies. After washing in PBS, sections were incubated with secondary goat anti-rabbit IgG for 20 min, followed by streptavidin for 20 min (Thermo Scientific, Wilmington, DE, USA). Peroxidase activity was visualized with 3,3-diaminobenzidine (DAB; Thermo Fisher, Wilmington, DE, USA), counterstained with Gill’s hematoxylin, washed, and mounted in Entellan^®^ (Merck, 1079610500, Darmstadt, Germany). Slides were examined under an Olympus BX51 microscope (Tokyo, Japan).

Brown precipitate in tissue sections was considered positive immunostaining. Immunoreactivity was evaluated blindly by three investigators. At high magnification (bar: 20 μm-X40), absence of staining was recorded as negative reaction (–), weak reaction as (+/–), while at low magnification (bar: 50/100 μm-X10/X20), positive staining was graded as strong reaction (+) or very strong reaction (++), based on the clarity and extent of cellular immunoreactivity.

### 2.6. Statistical Analyses

Data analyses were performed using the IBM SPSS 25.0 package program (SPSS^®^, Chicago, IL, USA). Graphs were generated using Microsoft Office Excel 2019 (Microsoft Corporation, Redmond, WA, USA). The Shapiro–Wilk test was used to assess the normality of data distribution. The Student t and Mann–Whitney U tests were performed for two-group comparisons. When comparing four groups, one-way analysis of variance (One-Way ANOVA) was performed for normally distributed data, and the Kruskal–Wallis test was performed for data that did not show a normal distribution. Pearson’s and Spearman’s correlation tests were used to identify potential correlations between the parameters. Tukey’s honestly significant difference (HSD) test, Tamhane’s test and the Mann–Whitney U test with the Bonferroni correction were used for pairwise comparisons (post hoc). The results are given as mean ± standard error of mean (SEM). Values of *p* < 0.05 were considered statistically significant.

## 3. Results

None of the dogs included in the study exhibited perioperative complications such as hemorrhage, incision site infection, or related disorders. All animals recovered uneventfully and were returned to their owners in good health. Furthermore, no clinical signs of pseudopregnancy were observed in any of the dogs in the DE group during the postoperative period.

### 3.1. Mean Ages and Body Weights of the Animals

There were statistically significant differences between the mean ages and body weights of the groups (Table 3).

### 3.2. Histomorphology of the Uterine Tissues and Microbiological Identification

All the tissue samples collected from the dogs with pyometra underwent histomorphological examination and bacterial identification. Accordingly, CEH was detected in all the samples collected from the pyometric dogs (14/14; 100%). Furthermore, *E. coli* was isolated from all the samples (14/14; 100%) belonging to the pyometra cases, but no bacteria were isolated from Groups DE and AE.

### 3.3. Serum Progesterone Concentrations

Serum P4 concentrations were significantly elevated in DE, CP, and OP groups compared to AE (between *p* = 0.004 and *p* = 0.044). The lowest level was observed in AE (0.52 ± 0.05 ng/mL), while the highest was in DE (16.63 ± 7.30 ng/mL).

### 3.4. Serum Anandamide and 2-AG Concentrations

**Figure 1:**
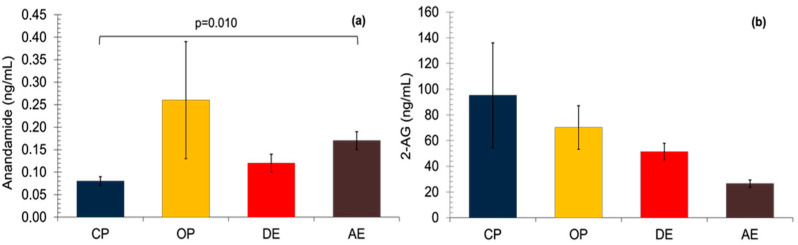
Serum ECB concentrations in pyometric and healthy dog (**a**) Serum AEA concentrations. A non-parametric Kruskal–Wallis test was applied followed by the Mann–Whitney U tests with Bonferroni correction. (**b**) Serum 2-AG concentrations. A non-parametric Kruskal–Wallis test was applied. Results are shown as mean ± SEM. *p* < 0.05 were considered significant. CP: closed-cervix pyometra, OP: open-cervix pyometra, DE: diestrus, AE: anestrus.

Serum AEA concentrations were highest in the OP group and lowest in the CP group, but statistically significant differences were observed only between the CP and AE groups (*p* = 0.010) (Figure 1).

The 2-AG concentrations were found to be lower from Group CP to Group AE, yet no statistically significant difference was determined between the groups (*p* = 0.072) (Figure 1).

### 3.5. Gene Expressions of CB1 and CB2

**Figure 2:**
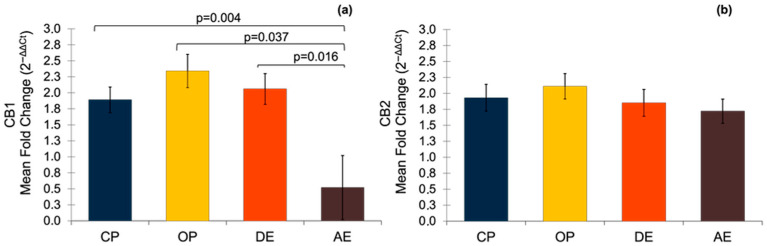
The mRNA expression of CBs in pyometric and healthy dog uterus. (**a**) Expression of CB1 gene. A parametric one-way ANOVA was applied followed by the Tukey HSD post hoc test. (**b**) Expression of CB2 gene. A parametric one-way ANOVA was applied. Results are shown as mean ± SEM. *p* < 0.05 were considered significant. CP: closed-cervix pyometra, OP: open-cervix pyometra, DE: diestrus, AE: anestrus.

It was determined that all of the target genes were expressed at different levels in all of the pyometra cases (CP and OP) and cyclic uterine tissues (DE and AE). The expression of CB1 significantly differed between the groups, such that it was significantly higher in the pyometra groups, and in the DE group compared to the AE group (Figure 2). On the other hand, no statistically significant difference was determined for the expression of the CB2 gene.

### 3.6. Localization of CB1 and CB2

**Figure 3:**
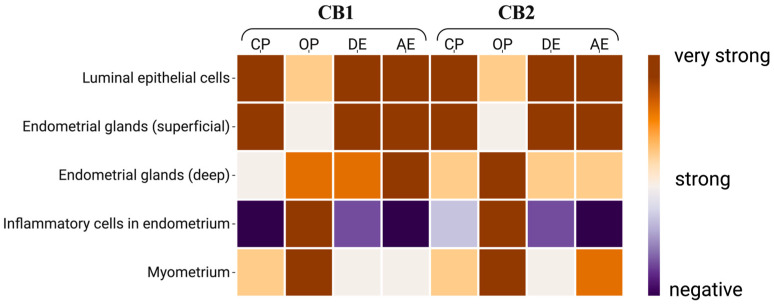
Heat map of CBs immunoreactivity at the cellular level in uterine tissue. CP: closed-cervix pyometra, OP: open-cervix pyometra, DE: diestrus, AE: anestrus.

Varying degrees of immunoreactivity for CB1 and CB2 were observed in the different layers of the uterus in all groups (Figure 3).

**Figure 4:**
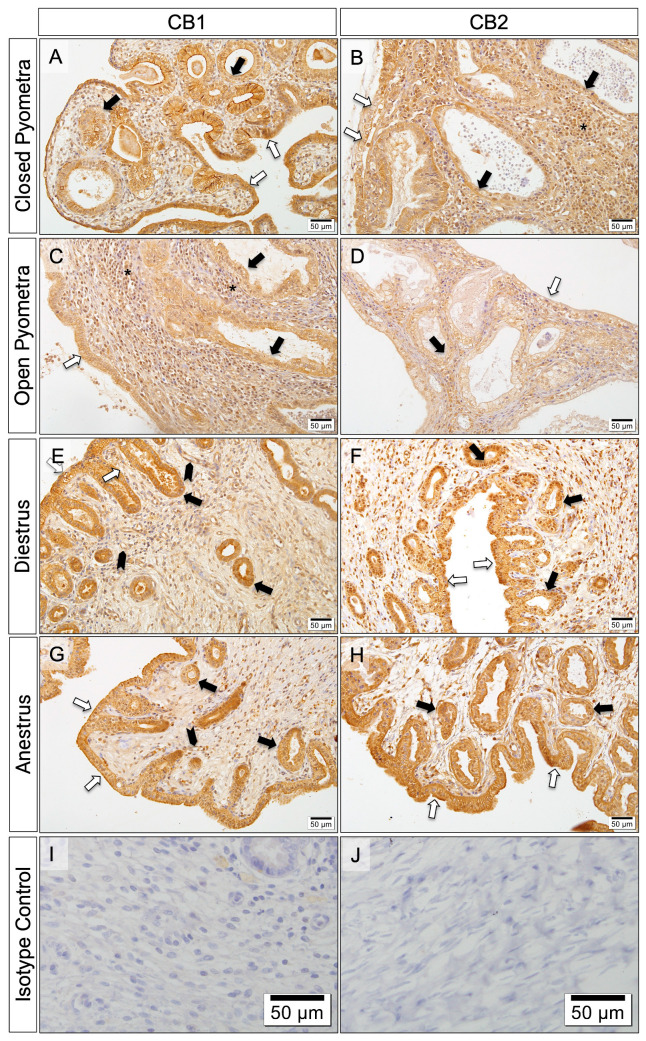
Immunolocalization of CBs in uterine tissue (bar: 50 μm-×20). Immunoreactivity of CB1 in different areas of the uterus by groups; CP (**A**), OP (**C**), DE (**E**), AE (**G**). Immunoreactivity of CB2 in different areas of the uterus by groups; CP (**B**), OP (**D**), DE (**F**), AE (**H**). No immunoreactivity in isotype controls (**I**,**J**). White arrows: luminal epithelial cells; black arrows: endometrial gland cells; *: immune cells.

Figure 3 shows a decrease in CB1 immunoreactivity from the surface to the depths of the uterine tissue in the CP, DE, and AE groups, a pattern not observed in the OP group. Immunoreactivity for CB1 was very strong in the luminal epithelial cells and endometrial gland cells of the uterus in the CP, DE and AE groups. It was also very strong in the immune cells and myometrium of the OP group. While CB1 immunoreactivity in the deep uterine glands was weak in Group CP, it was strong or very strong in the other groups. On the other hand, there was similar CB2 immunoreactivity in all the uterine layers in all of the groups (Figure 4).

## 4. Discussion

To the best of our knowledge, this is the first study to demonstrate the presence of the eCS in the canine uterus. The majority of previous investigations on the relationship between reproductive physiology or pathology and the eCS have focused on women or experimental animal models. In women, marked increases in glandular epithelium and endometrial vascular endothelial cells occur during the luteal phase of the menstrual cycle [23], which corresponds to the DE period in dogs [24]. Although endometriosis and pyometra represent two different conditions, both share the involvement of estrogen and P4 in their etiology [25]. Owing to their shared environment, comparable medical care, and similar aging patterns, dogs also serve as a valuable translational model for several human diseases, including reproductive disorders [26]. Accordingly, comparisons of eCS alterations between women and dogs appear well justified.

Serum ECB concentrations are influenced by multiple factors, including nutrition, physical activity, sex hormones, age, inflammation, and pregnancy [27]. In the present study, nutrition and physical activity were negligible, as all animals were companion dogs maintained on commercial diets. Thus, our discussion focuses primarily on the influence of hormonal status, decidualization, age and inflammation.

### 4.1. Effect of Hormonal Status

Serum P4 concentrations were within the expected physiological range in all groups, being elevated in the pyometra cases and during DE and at basal levels during AE. The influence of sex hormones on the eCS remains controversial. For example, in women with endometriosis, luteal-phase AEA concentrations are higher compared to both the follicular phase of endometriotic women and the luteal phase of healthy controls [12]. Higher CB1 expression has been reported in the fallopian tubes of women during the luteal phase, with P4 treatment inducing further upregulation [10]. However, other reports found no difference in AEA or 2-AG concentrations between the follicular and luteal phases of healthy women [12] and no effect of P4 on circulating ECB concentrations [28]. Uterine CB1 expression in women has been reported to be at low levels during the follicular phase [29].

The present study also highlights this complexity, as serum 2-AG concentrations were determined to have increased in the P4-dominant groups, while AEA concentrations had not. This discrepancy may be explained by two factors: (i) differences in the biosynthetic and degradation pathways of these mediators [6,7] and (ii) the effects of pre-analytical handling, including blood collection, storage, and processing [30]. Although the validity of the measurement method used here was previously confirmed [30], it has been reported that AEA and 2-AG concentrations may increase during the interval between blood collection and serum separation, even when samples are kept on ice. Moreover, prolonged storage at –80 °C appears to preserve 2-AG but not AEA, which continues to increase over time. Therefore, serum AEA and 2-AG are unlikely to serve as reliable clinical biomarkers [31]. Additionally, serum concentrations do not necessarily reflect tissue levels, as shown in reproductive tissues [32].

In our study, CB1/2 mRNA expression was numerically or statistically higher in the P4-dominant groups, suggesting that P4 may upregulate CB1/2 expression despite the absence of a direct correlation.

In this context, decidualization requires consideration, as it is a P4-driven process critical for the establishment of pregnancy [33]. Unlike in humans, decidualization in dogs is known to be embryo-dependent [34]. Nonetheless, decidual-like changes, such as PEH, are observed during canine diestrus/pseudopregnancy [35]. Moreover, some authors have described CEH as a deciduoma, reflecting a proliferative response that mimics decidual implantation sites [4,35].

The expression of eCS components has been demonstrated at both the mRNA and protein levels during decidualization and tissue remodeling in women and rats, although its precise role remains unclear [10]. The upregulation of CB1 mRNA has been shown in human [10] and rat decidua [32,36], and immunoreactivity for CB1/2 and other eCS proteins has been observed in human decidua [37]. These findings suggest a potential link between CB1 upregulation and peak decidual development [36]. This could explain the CB1/2 mRNA expression patterns observed in our DE group. Given that all nonpregnant bitches in DE are physiologically pseudopregnant, regardless of clinical signs [38], we propose that the increased CB1/2 expression detected in this study may be related to pseudoplacentational changes occurring during pseudopregnancy. We recognize that our interpretation above is speculative because we did not conduct a histological evaluation of PEH in the DE group. However, we believe that the increasing number of publications on PEH and their supporting data strengthen our speculation.

### 4.2. Effect of Age

In this study, the dogs in the pyometra groups were significantly older and heavier than those in the control groups. This finding is not unexpected, as pyometra is an age-associated pathology, whereas dogs in the control groups had been electively neutered at a young age. Nevertheless, age itself should be considered a critical contributing factor for the results obtained in the pyometra groups.

Throughout life, endometrial tissue undergoes continuous cycles of degeneration and regeneration under the influence of the HPO axis, referred to as endometrial plasticity [39]. A fundamental component of this plasticity is endometrial cell migration, which plays a central role in wound closure, prevention of excessive fibrosis, and facilitation of embryonic trophoblast invasion [40].

Stromal aging, defined as the irreversible arrest of the cell cycle [41], has been documented in canine CEH [42] and disrupts endometrial elasticity, thereby increasing susceptibility to pathological changes [43]. The high recurrence rate of CEH despite medical management further supports the concept of irreversible alterations in stromal cell cycling. In the present study, CEH was observed in all pyometra cases, underscoring its relevance in this context.

Components of the eCS exhibit dynamic, phase-specific regulation throughout the estrous cycle. In mice, eCS expression varies across the prepubertal, adult, late reproductive, and post-reproductive stages, reflecting both age- and organ-specific modulation in the endometrium [44]. The metabolic enzymes NAPE-PLD, FAAH, and DAGL-β exhibit the highest expression levels in the ovarian, oviductal, and uterine tissues, and these levels gradually increase with advancing age [44].

No significant differences were observed in CB1 mRNA expression in the uterine tissue between aged animals with pyometra and young animals in the DE and AE groups. These findings suggest that age alone may not directly influence CB1 or CB2 expression. Nevertheless, the potential impact of cellular senescence cannot be overlooked, as senescent cells persist within the uterus. The physiological and pathophysiological factors driving uterine cellular senescence, and their relationship with the eCS, need to be elucidated.

### 4.3. Inflammatory Status

Previous studies have demonstrated that TLR-2 and TLR-4 mRNA are involved in the uterine immune response to *E. coli* [5,45]. Specifically, uterine TLR-4 transcription is upregulated 2.4-fold in cases of pyometra compared to the DE phase of the estrus cycle [45]. Crosstalk between TLR-4 and ECB signaling has also been reported in the uterine tissue. In the mouse uterus, LPS increases CB1 mRNA expression while decreasing CB2 expression [14], and the pro-apoptotic effects of LPS are abolished by a CB1 agonist in pregnant mice [46]. Endocannabinoid system components are integral to macrophage-mediated inflammatory responses to TLR-4 stimulation [47], with macrophages being the main producers of ECBs [13]. Consequently, TLR-mediated macrophage activity modulates CBs and ECB expression, as evidenced by the LPS-induced upregulation of CB1 in mouse macrophages [47]. In contrast, LPS-induced CB2 expression in rat macrophages is controversial, with studies reporting both increases [48] and decreases [49]. Collectively, CB1 appears to inhibit TLR-4-mediated cytokine production [11], while CB2 may antagonize TLR-4 signaling [50]. These mechanisms likely underlie the CB1/2 mRNA expression observed in the pyometra groups, whereas the expression patterns in the DE and AE groups cannot be fully explained by LPS stimulation alone.

Toll-like receptor expression is also present during the DE and AE phases, with higher levels being observed during DE [5]. Two potential factors may contribute. Firstly, TLR-4 can be activated by sterile inflammation [51], which may explain its increased expression during DE. During DE, stromal leukocyte numbers are elevated [52], likely contributing to TLR-4 expression and suggesting coordinated endometrial defense against microbial invasion [53]. Secondly, TLR signaling regulates apoptosis via anti-apoptotic proteins or inhibitors, consistent with apoptosis occurring during DE [5]. Nonetheless, these factors alone do not explain CB1/2 expression during AE, when CB1 is low and CB2 is high. The elevated CB2 expression may reflect leukocyte activity, as CB2 is strongly expressed in both human and canine leukocytes, despite their low numbers in the uterus during AE [54].

Cyclooxygenase-2 expression is inducible by inflammatory stimuli, growth factors, and pathological conditions [55]. It is overexpressed in pyometric uteri, compared to the healthy uterus [6], but this does not lead to increased myometrial contractions, likely due to elevated serum P4 concentrations. This suggests that high PG expression in pyometra is linked primarily to inflammation [45]. Additionally, COX-2 may interact with the ECB system, as COX-2 has substrate affinity for ECBs [56], and ECBs exert potent relaxant effects via CB1, and to a lesser extent CB2, in both women [57] and mice [58]. Cyclooxygenase-2 is also highly expressed in the luteal endometrium of women [23], rats [59], and dogs [24]; contributing to physiological endometrial adaptation and tissue homeostasis. Notably, the upregulation of COX-2 and PGE synthase (PGES) in pyometra cases, and during the DE and AE stages coincides with TLR-2/4 upregulation, and LPS stimulation synchronously increases COX-2 and mPGES-1 expression, enhancing PGE2 synthesis [60]. Prostaglandin E2 exerts immunosuppressive effects on lymphocytes, monocytes/macrophages, and polymorphonuclear cells [61].

Immunohistochemically, TLR-4 localizes to different endometrial compartments in association with leukocyte populations, cytokines, and sex hormones. In cases of pyometra, the surface epithelium exhibits higher TLR-4 expression than the stroma, glandular epithelium, or healthy uterus. During DE and AE, the glandular epithelium expresses TLR-4 more intensely than the other compartments, with lower expression during AE than DE [5]. Cyclooxygenase-2 shows a similar distribution, suggesting parallel activation by LPS [24]. In this study, CB1/2 were immunolocalized to similar endometrial regions across all groups, mirroring TLR-4 and COX-2 localization and intensity, which can be shown as evidence for the cooperative regulation of the inflammatory response by ECBs, TLR-4, and COX-2.

Interestingly, in the OP group, CB1 and CB2 were more intensely expressed in the deep endometrial cells, inflammatory cells, and myometrium. This likely reflects OP-specific gene expression changes. Voorwald et al. (2015) [3] reported the overexpression of fatty acid-binding protein 3 (FABP3) in OP, a protein abundant in myocytes and vascular endothelium that is upregulated by LPS and can exacerbate inflammation [3]. Fatty acid-binding proteins also function as chaperones for ECBs, with ECBs exhibiting pronounced affinity for FABP3 [62].

## 5. Conclusions

In conclusion, this study provides the first direct evidence of ECB presence in both pyometra-affected and healthy canine uterine tissue, unequivocally supporting our initial hypothesis. The differential expression of CB1 and CB2 appears to result from the integrated effects of P4, decidualization, stromal aging, LPS, TLR signaling, and COX activity, reflecting the intricate crosstalk between reproductive physiology, uterine pathology, and the eCS. These findings not only advance our understanding of ECB regulation in the canine uterus, but also emphasize the need for further comprehensive investigations of additional components, including FAAH, NAPE-PLD, MAGL, and DAGL, at both tissue-specific and molecular levels to unravel their precise roles in uterine homeostasis and pathophysiology.

## Figures and Tables

**Table 1:** List of primers used for the RT-qPCR.

Gene	Primer Sequence	Product Length (bp)	Accession Numbers
CNR1	F: TCCTGGGGAGCGTCATATTT	99	AY011618.1
	R: TGACCCCACCCAGTTTGAAA		
CNR2	F: TCCTGGCCAGTGTGATCTTT	119	NM_001284480.1
	R: AGAGGCTGTGAAGGTCATGG		
KDM4A	F: TCACAGAGAAGGAAGTTAAG	82	XM_005629107
	R: TCACAGAGAAGGAAGTTAAG		
PTK2	F: ACCTGGCTGACTTCAATC	85	XM_005627993
	R: ATCTTCAACTGTAGCATTCCT		
EIF4H	F: TAAGGTCTCAGCAATTAC	101	XM_014114129
	R: TAAGGTCTCAGCAATTAC		

**Table 2:** Conditions employed for all antibodies during immunohistochemical procedures.

Antibody	Reference (Company)	Biological Source	Antibody Dilution	Antibody Incubation	Epitope Revelation (DAB)
CNR1/CB1	bs-1683R (Bioss, Woburn, MA, USA)	Rabbit (Polyclonal)	1:200	Overnight (17 h), 4 °C	Thermo Fisher Scientific Lab Vision Corporation
CNR2/CB2	LS-A34-50 (Life Span, Lynnwood, UT, USA)				
Isotype control	IgG (Vector Laboratories, Burlingame, CA, USA)	Rabbit	Same protein concentration as primary antibody	-	-

**Table 3:** Mean age and body weight of the groups. The data given as Mean ± SEM.

Groups	*n*	Ages (year)	Body Weight (kg)
CP	7	8.1 ± 1.1 ^a^	18.4 ± 1.2 ^a^
OP	7	10.0 ± 1.0 ^a^	15.5 ± 0.1 ^a^
DE	7	1.8 ± 0.1 ^b^	10.1 ± 0.9 ^b^
AE	7	1.5 ± 0.2 ^b^	11.2 ± 1.0 ^b^
*p*		<0.001	<0.001

CP: closed-cervix pyometra, OP: open-cervix pyometra, DE: diestrus, AE: anestrus. Values within the same column followed by different letters (^a,b^) are significantly different.

## Data Availability

The original contributions presented in this study are included in the article. Further inquiries can be directed to the corresponding author.
